# Sternal Hardware Migration Following Rigid Plate Fixation After Coronary Artery Bypass Graft

**DOI:** 10.1016/j.atssr.2025.09.004

**Published:** 2025-10-03

**Authors:** Hao Huang, Craig R. Smith, Jeffrey A. Ascherman

**Affiliations:** 1 Division of Plastic Surgery, Department of Surgery, NewYork-Presbyterian, Columbia University Irving Medical Center, New York, New York; 2 Division of Cardiac Surgery, Department of Surgery, NewYork-Presbyterian, Columbia University Irving Medical Center, New York, New York

## Abstract

Rigid plate fixation is becoming an increasingly popular option for sternotomy closure because some surgeons have reported better sternal healing and lower rates of sternal complications. This case report discusses the rare complication of hardware migration after rigid fixation using the SternaLock Blu system (Zimmer Biomet). A high index of suspicion and multidisciplinary team collaboration are important for the early recognition and management of this complication.

Median sternotomy is the most frequently used incision in cardiothoracic surgery. There are many methods for sternal closure after sternotomy, with wire cerclage the most common. SternaLock Blu (Zimmer Biomet), a commercially available rigid plate fixation system, uses titanium plates of various configurations and self-drilling and self-tapping screws.^1^^,^^2^ In a prospective, multicenter randomized controlled trial, SternaLock fixation had been shown to result in significantly better sternal healing, higher rates of sternal union, and lower rates of sternal complications compared with traditional wiring.^1^

Although shifting, bending, breaking, or loosening of plates and screws is cited as a possible risk of the SternaLock system, no studies or reports in the literature have mentioned such a complication in the context of sternal reconstruction. We present a case of hardware failure after rigid plate fixation in a patient who underwent coronary artery bypass grafting (CABG).

A 71-year-old man with a history of hypertension, hyperlipidemia, diabetes, atrial fibrillation, elevated body mass index (30.4 kg/m^2^), and coronary artery disease underwent 3-vessel CABG and median sternotomy closure with interrupted wires. Four months after CABG, he presented with a 1-month history of worsening sternal pain, clicking, and instability. Operative exploration revealed sternal dehiscence and fracture of all sternal wires. After the wires were removed, the sternal halves were reapproximated with the SternaLock system: one 6-hole plate was fixed across the manubrium with screws, and one 6-hole plate and one 4-hole plate were fixed to the sternal body, also with screws. There were no intraoperative issues with plate implantation. To facilitate bony healing, bilateral pectoralis major myocutaneous flaps were advanced to the midline over the plates before closure.

Four months after the second operation, the patient began to experience anterior chest wall pain, swelling, and occasional serous drainage through a pinpoint opening in the sternotomy incision. The collection was aspirated under sterile conditions, and the serous fluid was sent for analysis. Results of culture were negative, and the triglyceride level was low (60 mg/dL), ruling out a possible chyle leak. Computed tomography (CT) of the chest (Figure 1) was notable for a right anterior thoracic wall simple collection measuring 9 cm × 5 cm adjacent to the sternotomy site and extending to the level of the skin. On the basis of an interdisciplinary discussion among cardiac surgery, plastic surgery, and radiology, at least 1 of the plates did not appear adherent to the sternum on CT, thus potentially causing irritation to surrounding tissues and accounting for the delayed seroma formation. Displaced screws were evident on both the CT scan and on a recent shoulder roentgenogram (Figure 2), which was obtained separately for evaluation of shoulder pain.

**Figure 1:**
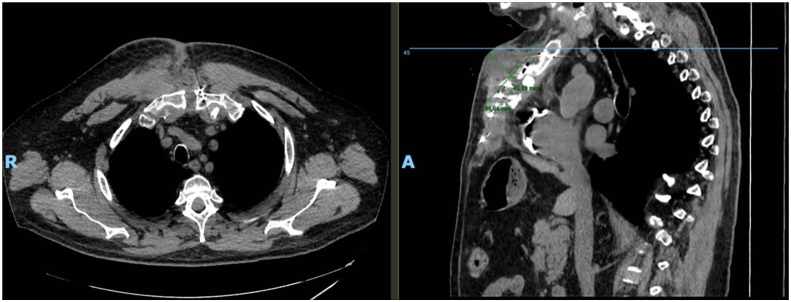
Axial and sagittal computed tomographic views of the chest that show a right anterior chest wall collection. (A, anterior; R, right.)

**Figure 2:**
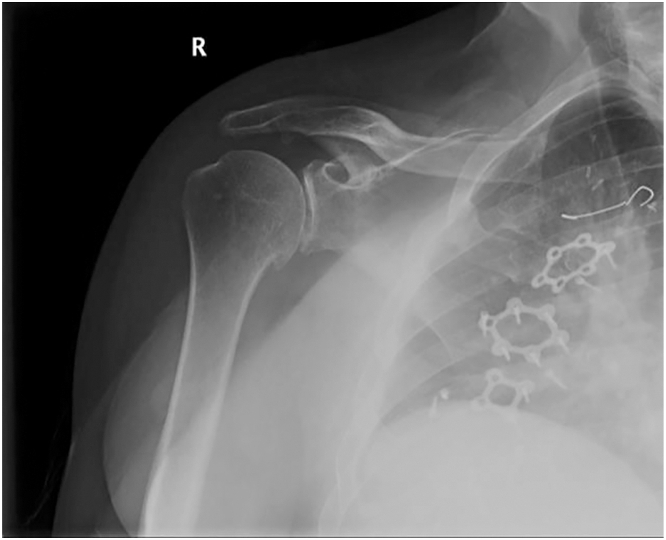
Anteroposterior view of a right shoulder roentgenogram that incidentally detected displaced screws and potentially displaced plates. (R, right.)

The decision was made to return to the operating room. On opening the previous incision, we encountered and removed several free-floating screws. Using fluoroscopy, we identified the remaining screws in the subcutaneous tissue, pectoralis flaps, and chest wall, and some of these screws had migrated as far laterally as the midclavicular line (Figure 3). A few screws had remained attached to the plates, whereas most had either loosened or become completely displaced. All 3 plates had loosened from the sternum. No plates were fractured. Intraoperatively, there were no gross signs of infection (culture results were also negative). The pectoralis flaps were readvanced to the midline before closure. In total, all 3 plates and 16 screws were removed. Final intraoperative fluoroscopy films demonstrated removal of all sternal hardware.

**Figure 3:**
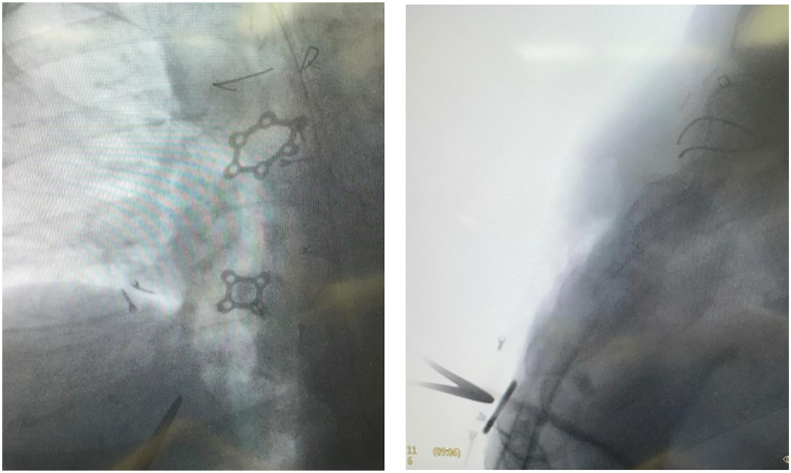
Intraoperative fluoroscopic images that show displaced screws as far laterally as the midclavicular line.

Although the patient recovered well initially, his course was later complicated by *Candida*-positive sternal wound infection and osteomyelitis requiring bony debridements, pectoralis flap readvancement, and an omental flap for coverage with additional vascularized tissue. He received intravenous antifungal treatment postoperatively as well as close follow-up with infectious disease and plastic surgery.

## Comment

Wire cerclage is effective in approximating the bony halves after median sternotomy and providing relative stability. However, some surgeons believe that it does not confer rigid fixation.^1^ Rigid plate fixation of the sternum has been shown to have superior mechanical stability in in vitro studies^3^ and to lead to better clinical outcomes in some studies.^4^ The SternaLock Blu system, which is one type of plate fixation, has been used with promising results in sternotomy closure as well as in complex sternal reconstruction for anterior chest wall deformities.^1^^,^^5^ In reviewing the literature, we did not find any reports of SternaLock hardware failure in sternal reconstruction. Conversely, hardware failure has been described in the fixation of traumatic rib fractures in as many as 10.9% of cases, with screw loosening the most common cause of failure.^2^ We present a case of hardware migration after sternotomy closure using SternaLock plate fixation.

Although it is challenging to pinpoint the cause of the hardware failure, the biomechanics of the sternal segments after sternotomy likely played a significant role. For rib fractures, it is known that the posterolateral region of the chest wall experiences the most mechanical stress during respiration, and this contributes to fixation plate fatigue.^2^ Our patient’s previous history of sternal dehiscence and wire breakage suggests that he likely had unfavorable sternal mechanics for bone healing. Unlike fixation of long bones, immobilization after sternotomy closure to counteract micromovements across the osteotomy is not possible, thereby allowing for propagation of unfavorable forces through the hardware during movement. The patient’s history of recurrent hardware failure and the finding that all 3 SternaLock plates had failed make technical errors less likely contributing factors. Other factors, such as infection, are also unlikely given the sternum’s healthy appearance intraoperatively.

Our patient represents a particularly high-risk patient who possibly could have benefited from enhanced sternal closure. The new SternaLock 360 system combines rigid plates with flexible sternal bands, and it has reduced sternal edge motion and low rates of sternal complications in high-risk cardiac patients.^6^ Furthermore, a longitudinal fixation system could have been helpful in redistributing forces along the length of the sternum, thereby mitigating forces across transversely placed hardware. In their 10-year experience, Madjarov and colleagues^7^ found decreased rates of deep wound infections and 30-day mortality in patients with longitudinal rigid fixation, although hardware failure was not reported as an outcome.

This case underscores the rare complication of hardware failure after SternaLock plate fixation in sternotomy closure. It is important to have a high index of suspicion for this complication in patients presenting with sternal pain and instability, as well as delayed seroma formation in the absence of infection. Patients at high risk for hardware failure may benefit from enhanced closure systems, such as longitudinal rigid fixation. More studies are needed to elucidate risk factors for hardware failure after rigid sternal fixation with plates.
